# Providers’ Initial Trust on an Organization-Sponsored Sharing Platform: The Framing of Coworker Collaborative Consumption

**DOI:** 10.3389/fpsyg.2020.02174

**Published:** 2020-09-08

**Authors:** Anita D. Bhappu, Kirsimarja Blomqvist, Tatiana Andreeva, Paola Zappa, M. Lisa Yeo, Tea Lempiälä

**Affiliations:** ^1^School of Engineering, University of California, Merced, Merced, CA, United States; ^2^School of Business and Management, LUT University, Lappeenranta, Finland; ^3^School of Business, Maynooth University, Maynooth, Ireland

**Keywords:** initial trust, cognitive heuristics, coworker trustworthiness, social exchange, two-sided platforms, goods and services, mobile apps, sharing economy

## Abstract

Organization-sponsored sharing platforms extend the sharing economy to workplaces by connecting employees in a private online community where they can socially exchange goods and services with coworkers. Employees share costs but do not earn income during this collaborative consumption. Furthermore, employers pay for their employees to have access to the platform technology and any related transaction fees. Trust is a crucial antecedent for engagement on sharing platforms because it helps mitigate risks during collaborative consumption. However, the literature on trust in the sharing economy has focused almost exclusively on platforms that broker peer-to-peer rental transactions rather than social exchanges. There is also a lack of research about providers’ perspectives. We address these gaps by investigating the nature of trust among employees who initially provide goods and services on an organization-sponsored sharing platform. We also explore how these employees’ initial trust influences their collaborative consumption with coworkers. Through abductive analysis of 22 interviews with 15 providers on an organization-sponsored sharing platform, we shed light on how employees initially develop trust when providing goods and services to coworkers. By integrating prior research on initial trust among employees and cognitive framing with in-depth qualitative insights, we develop a conceptual model depicting how identity, interaction and issue frames shape these providers’ beliefs about coworker trustworthiness and intended sharing strategy. In particular, our empirical findings reveal that employees’ social categorization, illusions of control and engagement motive framed their initial trust and enactment of collaborative consumption as citizens in a community or consumers in a marketplace.

## Introduction

Understanding how trust influences social relations has been the focus of cross-disciplinary research in interpersonal (Dietz, 2011) and organizational behavior (Dirks and Ferrin, 2001; McEvily et al., 2003). Within the field of organizational behavior, trust implies a willingness to assume risks and be vulnerable to the behavior of others (Mayer et al., 1995). It involves also a positive expectation that others will behave competently with goodwill (Blomqvist, 1997; Rousseau et al., 1998) and integrity (Mayer et al., 1995). Trust is essential for effective interpersonal collaborations (Lewicki et al., 1998) and particularly relevant in social exchanges that are characterized by interdependency and information asymmetry between actors (Rousseau et al., 1998). Interactions among peers who engage as consumers and providers in the sharing economy resemble this type of social embeddedness because they often involve interpersonal vulnerability during exchange of goods and services (Cho et al., 2007; Belk, 2010). Trust is, therefore, a crucial antecedent for engagement on a sharing platform, which is the digital technology that connects consumers and providers of goods and services online. Trust helps to overcome uncertainty and mitigate risks during facilitated rental transactions and social exchanges between them; this is called collaborative consumption (Botsman, 2010; Huurne et al., 2017).

The emerging literature on trust in the sharing economy builds on extensive research about trust in e-Commerce environments (McKnight et al., 2002; Gefen and Straub, 2004). Recent empirical findings by Hawlitschek et al. (2016) suggest that it is important to distinguish and account for what is being trusted – the peer, the platform or the product – during collaborative consumption, as well as the role of the trustor as a consumer or a provider in a given peer-to-peer sharing exchange. Using a similar framework, Huurne et al. (2017) conducted a systematic literature review of research on trust and peer-to-peer collaborative consumption. They conclude from a synthesis of 45 studies that trust is the most important antecedent of consumer engagement in the sharing economy. They also note, however, that there is a lack of research about trust from providers’ perspectives “although trust is likely to be just as important for them as they provide access to their assets” (Huurne et al., 2017, p. 494) and is essential to a “platform’s capability to generate activity … [especially] in case of unexpected turns or damages” by consumers (Hawlitschek et al., 2016, p. 32). Furthermore, this nascent body of research on trust in the sharing economy has focused almost exclusively on sharing platforms that broker peer-to-peer rental transactions (e.g., Airbnb) rather than platforms for social exchanges (e.g., Couchsurfing) (Huurne et al., 2017).

Our research offers insights about providers on an organization-sponsored sharing platform that connects employees in a private online community where they can socially exchange goods and services with coworkers (Bhappu and Schultze, 2018). As such, organization-sponsored sharing platforms represent the “sharing economy ideal” (Acquier et al., 2017) because they integrate all three core elements of the sharing economy, namely access, platform and community. Examples of organization-sponsored sharing platforms include Scoop^®^ and Zimride^®^, which a number of companies and universities use to promote ridesharing among their employees to and from work, as well as Rheaply^®^, which facilitates employee sharing of organizational assets such as lab and office equipment. On these organization-sponsored sharing platforms, employees share costs but do not earn income from collaborative consumption. Their social exchanges are “built on different trust mechanisms, such as a sense of community, intrinsic motivation of participants, and social norms and values” (Huurne et al., 2017, p. 495). Furthermore, employers pay for their employees to have access to the platform technology and any related transaction fees. Hence, in comparison to sharing platforms such as Uber^®^ or Airbnb^®^ that facilitate peer-to-peer rental transactions, an organization-sponsored sharing platform offers a particularly useful setting for investigating providers’ trust during social exchanges.

Specifically, we investigate the following research questions: *What is the nature of trust among employees who initially provide goods and services on an organization-sponsored sharing platform? How does it influence the way these providers perceive and enact collaborative consumption with coworkers?* We, therefore, begin this paper by reviewing and integrating the literature on trust among employees, as well as on cognitive framing and trust in the sharing economy, in order to conceptually ground our research. Next, we describe the site of our field study, sharing platform, data collection and analysis. We then describe qualitative insights from our provider interviews and theorize them to develop our conceptual model for providers’ initial trust on an organization-sponsored sharing platform. Finally, we discuss the contributions and limitations of our research, including suggestions for future research.

## Conceptual Development

### Trust Among Employees

Trust is a multi-faceted and complex concept (Blomqvist, 1997; Castaldo et al., 2010). A large body of psychological, sociological and management research (e.g., Roberts and O’Reilly, 1974; Cook and Wall, 1980; Brockner et al., 1997; Mayer and Davis, 1999; Tan and Lim, 2009) has demonstrated that trust is essential for interpersonal workplace interactions. Trust among employees is an antecedent of workplace relationships because it affects an employee’s choice of social exchange partners (Granovetter, 1985; Uzzi, 1996, 1997) and willingness to engage in risky behavior such as knowledge sharing (Bakker et al., 2006), cooperation (Dirks and Ferrin, 2001) or organizational citizenship behavior (McAllister, 1995).

In our research, we define trust as “a psychological state comprising the intention to accept vulnerability based upon positive expectations of the intentions or behavior of another” (Rousseau et al., 1998, p. 395). To understand how employees who initially provide goods and services on an organization-sponsored sharing platform come to trust coworkers whom they have not yet met, we build on McKnight et al.’s (1998) model of initial trust in new organizational relationships. It conceptually accounts for both the employee (trustor) and a coworker (trustee) whose trustworthiness they are evaluating, as well as the situational context of their organizational relationship. It depicts the development of initial trust as an interplay of an employee’s disposition to trust, cognitive processes and institution-based trust, which influence their trusting beliefs about a coworker and trusting intentions toward them.

Trust formation in a new relationship is a gradual interpersonal process wherein two individuals who have no prior experience interacting together learn to depend on each other over time (Mayer et al., 1995; Lewicki et al., 2006; McKnight and Chervany, 2006; Wilson et al., 2006; van der Werff and Buckley, 2017). When this process first begins, both individuals have to decide whether to initially trust the other. Researchers (Frazier et al., 2013; Colquitt et al., 2007) have discerned that individuals have a disposition to trust, which by definition consists of having “faith in humanity” – a relatively stable personality trait (see Rotter, 1967; Wrightsman, 1991) and a “trusting stance” – a more calculative decision or choice to trust because one believes that trusting others facilitates success (see McKnight et al., 1998). Some individuals may have a more trusting personality (Reimann et al., 2017) and choose to trust before they get to know another individual interpersonally because they believe that trusting individuals pays off in most situations (McKnight et al., 1998). Therefore, disposition to trust is particularly important for the development of initial trust when individuals have no prior experience interacting together and have to make cognitive and affect-based judgments about the trustworthiness of others (Colquitt et al., 2007).

When forming beliefs about coworker trustworthiness, individuals evaluate a coworker’s ability, benevolence, integrity and predictability (Mayer et al., 1995; McKnight et al., 1998) using available information. However, beliefs about coworker trustworthiness evolve over time as more or different information about their characteristics and behavior becomes available (McKnight and Chervany, 2006; Wilson et al., 2006). Individuals’ trusting beliefs are influenced by their cognitive processing of a coworker’s reputation, as well as their own social categorization and illusions of control in interactions with them (McKnight et al., 1998). Cognitive processing implies that “trust relies on rapid, cognitive cues or first impressions, as opposed to personal interactions” (McKnight et al., 1998, p. 475). By making a conscious judgment about whether and whom to trust (e.g., Brewer, 1981; Meyerson et al., 1996), individuals make a ‘leap’ of inference about coworker trustworthiness going “beyond the expectations that reason and experience alone would warrant” (Lewis and Weigert, 1985, p. 970). If the individual concludes that a coworker has a good reputation, or is part of the same social group as themselves, then they will consider this coworker to be more trustworthy than other employees. If an individual believes that organizational relationships are predictable and monitored, then they will also be more trusting of coworkers. Employees’ trusting beliefs, in turn, influence their willingness to depend on and be vulnerable to unknown coworkers (Currall and Judge, 1995). Organizational routines and governance can also enhance employees’ trusting beliefs and intentions because they represent institution-based trust and provide safeguards against non-normative and deviant coworker actions (Lewis and Weigert, 1985).

### Cognitive Framing of Providers’ Initial Trust

When an employee lists items that they are willing to share with coworkers on an organization-sponsored sharing platform, they typically do not know who will request access to their provided goods and services. Research on the sharing economy suggests that trust is particularly important for individuals who “provide access to their assets” (Huurne et al., 2017, p. 494) because it helps them overcome uncertainty and risk associated with collaborative consumption (Botsman, 2010; Huurne et al., 2017), which may make providers feel particularly vulnerable and cause them to actively assess situational risks (Cho et al., 2007; Belk, 2010). Providers’ initial trust can, therefore, increase intentions to share their assets (Hawlitschek et al., 2016) and decrease inhibitions for unknown consumers to access them (Chiles and McMackin, 1996; Nooteboom, 2016). For these reasons, the development of employees’ initial trust as providers on an organization-sponsored sharing platform is crucial. In the absence of information about the identity and reputation of potential social exchange partners, employees’ initial trust should be influenced by cognitive processes such as social categorization and illusions of control (McKnight et al., 1998).

Social categorization is fundamental to how individuals enact relationships with others (Tajfel and Forgas, 2000; Dewulf et al., 2009). Three prior studies (Strader and Ramaswami, 2002; Lu et al., 2010; Malinen and Ojala, 2013) within the sharing economy literature find a positive effect for familiarity among social exchange partners on trust and sharing behavior. “The influence of familiarity may be explained by the concept of perceived similarity (Lu et al., 2010), also referred to as homophily. It points to the mechanism whereby trust is based on common characteristics between the trustor and the trustee” (Huurne et al., 2017, p. 492), which are known to increase sharing behavior (Zellmer-Bruhn et al., 2008). Unlike complete strangers transacting peer-to-peer market exchanges (Schor, 2016), research suggests that individuals on an organization-sponsored sharing platform perceive themselves as a community and assume social exchange partners will act benevolently (Bhappu and Schultze, 2018). Therefore, an employee’s social categorization of themselves and coworkers as members of the same organizational community could facilitate their assumption that potential social exchange partners are trustworthy until proven otherwise (Chen et al., 2009). The latter is an example of “unit grouping” and “stereotyping,” which are two of the social categorization processes that impact initial trust (McKnight et al., 1998).

Similarly, employees may have illusions of control (Langer, 1975; McKnight et al., 1998) about their social exchanges with coworkers, especially when they have had no prior interactions (Ferrin and Dirks, 2003). In uncertain and unfamiliar situations, individuals inappropriately assume that they can personally control how events unfold because this assumption makes them feel more confident that they can avoid any negative outcomes (Langer, 1975). Research (Bhappu and Schultze, 2018) suggests that individuals on an organization-sponsored ridesharing platform incorrectly assume that community members will not take advantage of them (financially or physically) and that organizations will govern deviant individual behavior. Illusions of control may, therefore, enhance an employee’s initial trust when providing goods and services to coworkers. Employees may also take small actions – token control efforts – to test whether a coworker is trustworthy when they first meet them. “Token control efforts will give a person the illusion that his or her positive faith in humanity can apply to the individual” (McKnight et al., 1998, p. 481).

As described, illusions of control and social categorization function as cognitive representations or frames that influence trusting beliefs and intentions (Dewulf et al., 2009; Lewicki and Brinsfield, 2011). Identity frames shape how an employee conceives of themselves and their coworkers in a given social context. Interaction frames, on the other hand, provide scripts for normative interpersonal relationships between an employee and coworkers. Finally, substantive issue frames help employees assess the potential gains and losses when making a risky choice. In the case of an organization-sponsored sharing platform, an employee’s motive or goal for consuming collaboratively with coworkers also represents a substantive issue frame that can influence their trusting beliefs and intentions (Lewicki and Brinsfield, 2011). These cognitive frames are psychologically rooted in the four heuristics of representativeness, availability, anchoring and adjustment, and affect that bias individual decision-making (c.f. Tversky and Kahneman, 1981; Bazerman, 2001; Cornelissen and Werner, 2014). Therefore, the framing of providers’ initial trust in these different ways “will generally persist until influenced by a new experience; hence, the frame reduces the need for effortful monitoring and frequent reanalysis of a situation or relationship. Only when a relevant new experience passes a certain threshold of perceptual salience will the frame be adjusted” (Lewicki and Brinsfield, 2011, p. 3).

## Materials and Methods

The framing of employees’ initial trust when providing goods and services to coworkers offers a theoretical lens for answering our research questions, which we investigated by launching an organization-sponsored sharing platform at a public university in the United States. We now discuss this organizational setting and technology platform used in our field study, as well as our data collection and qualitative analysis, before describing and discussing our findings.

### Organizational Setting

The university agreed to pilot our organization-sponsored sharing platform because it has a strong commitment to campus sustainability and community engagement. In doing so, it also supported faculty research because three co-authors are employed at this university. Among the university’s approximately 1700 employees, 54% were female, 24% were faculty, 67% were staff, and 9% were other academic positions. In terms of ethnicity, the two biggest groups were White (50%) and Hispanic (23%). Our study was approved by the university’s Institutional Review Board and we obtained consent from participants to share their deidentified (redacted) interview quotes and survey responses in our research reporting and publications.

A university is an opportune setting in which to study the sharing behavior of employees for several reasons (e.g., Biancani et al., 2014). First, it is a heterogeneous work environment that includes employees with diverse demographic and work-related characteristics in various faculty and staff roles. A large university also has a complex organizational hierarchy consisting of many departments and functions across multiple buildings and locations, which resemble the matrix structures and organizational dynamics of large corporations.

### Technology Platform

On our organization-sponsored sharing platform, employees could engage in sharing exchanges with coworkers using two mobile applications. The *Share@Home* (S@H) mobile application facilitated employee sharing of goods and services for *personal* use. The *Share@Work* (S@W) mobile application facilitated employee sharing of goods and services for *professional* use. Both mobile applications recorded users’ logins, messages, and sharing exchanges, as well as any data that users inputted or deleted about their goods, services and profile. The mobile applications did not store any location or search data and did not have reputation or performance ratings.

To use either of the mobile applications, employees had to first authenticate themselves using the university’s single sign-on system, then accept our study consent form and finally agree to the technology provider’s terms of use. Once they did that, employees could set up a profile with their contact information; a profile photo was recommended but optional. No money was exchanged when lending goods or volunteering services via the mobile applications; all items were shared for free. To offer a good or service on the mobile applications, employees had to provide descriptive information and calendar availability for the item, and upload at least one photo. When deciding what to offer, employees could review and respond to coworkers’ posted needs or just list a good or service that they felt comfortable sharing. To find what they needed or to browse listed goods and services, employees could scroll through newly offered items or conduct an item search by keyword or category. They could anonymously message coworkers who were offering items that interested them; these coworkers were anonymous to them too. They could also post a need for a good or a service that was not currently offered.

When an employee submitted a request for a good or service using either of the mobile applications, the platform sent a push notification to the coworker who had offered to share it. This coworker could review the employee’s profile information, as well as their requested start and end dates plus exchange location. If the coworker denied their sharing request, the employee received a push notification of this decision but the coworker remained anonymous. If their sharing request was accepted, the employee received a push notification and gained access to the coworker’s profile information for coordinating the scheduled exchange. As sharing exchanges progressed over time, the platform sent both the employee and the coworker push notifications and status updates including reminders of scheduled meetups and prompts to confirm completed actions (e.g., good was picked up, service has started).

### Data Collection

**FIGURE 1:**
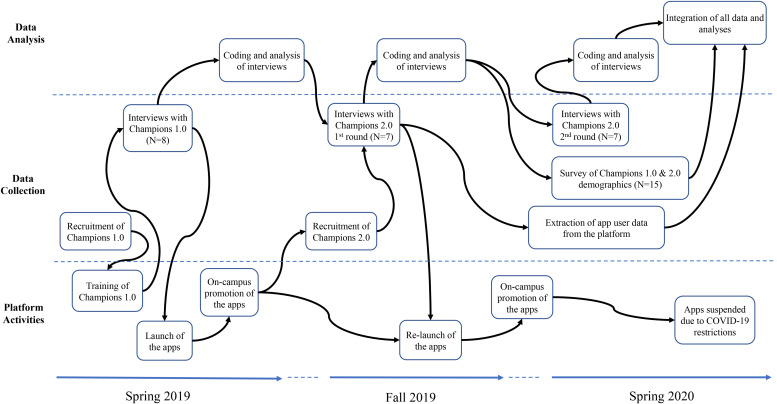
Overview of data collection.

We collected data for this paper as part of a longitudinal field study about our organization-sponsored sharing platform. The data consists of 22 interviews with 15 employees who agreed to provide goods and services when the S@W and S@H mobile applications were initially introduced at the university. An overview of our data collection is depicted in Figure 1.

Prior to the launch of the mobile applications in spring 2019, we invited via email several university employees to be a “community champion”; they had previously participated in a focus group or interview during the technology development phase of our field study. The primary role of community champions – who were compensated research participants – was to provide goods and services (initial inventory) on both mobile applications. Of the 29 invited employees, 8 (28%) responded and consented to being one (referred to as *Champions 1.0*). With our assistance, Champions 1.0 then had to install one or both applications on their mobile devices, as well as add three goods or services plus post one need. We reminded them to complete this task prior to their scheduled interview, which took place before the official campus launch of the applications (except for one that occurred immediately thereafter due to scheduling issues). The Champions 1.0 were, therefore, familiar with the technology by the time we interviewed them and could reasonably make sense of using it to consume collaboratively with coworkers. Most, but not all, of the Champions 1.0 added a variety of goods, services and needs prior to their interview. Those who had not completed these tasks had, however, considered what they were going to share and were able to discuss their thought process. Approximately 3 weeks after the official campus launch of the applications, we instructed all the Champions 1.0 to initiate a request for an available good or service on the applications; most of them did not complete this latter task.

For the campus launch of the applications in spring 2019, the university’s Chief Information Officer and Director of Sustainability jointly sent an official email to all employed faculty and staff introducing the mobile applications as part of a research study conducted by the first author. This email included hyperlinks for downloading the mobile applications from our study website, which also contained instructional videos on how to use the technology, animated videos of university use cases and frequently asked questions, as well as the end user agreement and study consent form. For a month after this email was sent, the research team promoted the mobile applications on campus by setting up an information table that rotated across all buildings (except student accommodation) and parking lots, as well as distributing fliers and business cards with QR codes to download the applications.

Analysis of platform user data revealed that 50 unique employees had registered to use one or both of the mobile applications during the 6 months after the official campus launch with 42 users on each of the applications. Therefore, our sample of Champions 1.0 represented 16% of the overall population of users at that time. During these 6 months, 50 items were offered on both applications (23 on S@W; 27 on S@H) and 14 needs were posted (6 on S@W; 8 on S@H). However, only 2 sharing exchanges were completed and 3 sharing requests timed out after 48 hours without a response (all on S@W).

In an effort to increase engagement on our organization-sponsored sharing platform, we decided to relaunch the mobile applications to university employees in fall 2019. To recruit a new set of community champions, we invited via email every employee who had registered to use the applications at that time to be a community champion except those who had been Champions 1.0. Of the 42 invited employees, 7 (16%) consented to be one (referred to as *Champions 2.0*). They were already experienced with the technology but became compensated research participants at that time. Similar to Champions 1.0, they were instructed to post three goods or services plus one need on the mobile applications prior to their interviews, which took place before the official campus relaunch email was sent out. The text of the official campus relaunch email sent to all employed faculty and staff was almost identical to the one sent during round one and the research team began similar promotional activities thereafter. Approximately three weeks later, we asked all the Champions 2.0 to initiate a request for an available good or service on the applications. Most of them completed all of these assigned tasks, unlike the Champions 1.0, possibly because they were early adopters of the mobile applications. Therefore, we decided to conduct an additional round of interviews with the Champions 2.0 to have them further elaborate on their engagement and make sense of their experiences on our organization-sponsored sharing platform. These second interviews were conducted approximately 90 days after the official campus relaunch email was sent.

At that point in time, which was approximately 10 months since the mobile applications were first launched, 75 unique employees had registered to use one or both of them (59 on S@W; 64 on S@H). Therefore, our combined sample of 15 community champions represented 20% of the overall population of platform users at that time. During these 10 months across both applications, 57 items were offered, 18 needs were posted, 5 sharing exchanges were completed and 7 sharing requests timed out. Together, Champions 1.0 and 2.0 were responsible for 90% or more of this user activity, which was split equally between the S@W and S@H mobile applications. To measure the self-reported demographic characteristics of all champions (see Table 1), we administered a short Qualtrics survey in spring 2020.

**TABLE 1:** Sample characteristics.

Champion group	ID	Number of interviews	University role	Tenure at university (years)	Age (years)	Sex	Ethnicity	Engagement motive	Initial provider trust
1.0	A	1	Faculty	1–3	25–39	Male	Hispanic	Citizen	High
1.0	B	1	Staff	>12	40–54	Male	White	Consumer	Moderate
1.0	C	1	Staff	10–12	40–54	Male	White	Citizen	High
1.0	D	1	Staff	10–12	40–54	Male	White	Consumer	Moderate
1.0	E	1	Faculty	4–6	25–39	Male	Hispanic	Citizen	Moderate
1.0	F	1	Staff	4–6	55–69	Female	White	Consumer	Low
1.0	G	1	Staff	>12	55–69	Male	Hispanic	Citizen	Moderate
1.0	H	1	Staff	n/a	40–54	Male	Asian	Citizen	Moderate
2.0	I	2	Staff	<1	<25	Male	Hispanic	Consumer	Low
2.0	J	2	Staff	4–6	25–39	Female	Black	Citizen	Moderate
2.0	K	2	Staff	<1	55–69	Female	White	Consumer	Moderate
2.0	L	2	Staff	1–3	25–39	Female	Hispanic	Consumer	Moderate
2.0	M	2	Staff	7–9	25–39	Female	Black	Citizen	High
2.0	N	2	Staff	10–12	40–54	Female	Hispanic	Citizen	High
2.0	O	2	Staff	7–9	25–39	Female	White	Consumer	Low
Total:	15	22							

### Qualitative Analysis

Our champion interviews each lasted about an hour, were audio recorded and later transcribed. Using cognitive framing as our analytical lens, our interview questions probed how they perceived collaborative consumption with coworkers, how they decided what goods and services to offer, the significance and value of these items, their perceived risks in offering these items on the S@W and S@H mobile applications, how they managed this vulnerability and their sharing behavior. Our semi-structured interview protocols can be found in the Appendix. During the interviews, we focused on understanding champions’ discursive explanations of collaborative consumption with coworkers, as well as their actions and experiences using our organization-sponsored sharing platform.

We adopted an abductive approach (Locke et al., 2008) to qualitatively analyze the champion interviews. Abduction is a process of iteratively going back-and-forth between theory and data to arrive at new insights that are both empirically and theoretically grounded (Van Maanen et al., 2007). We inductively developed initial hunches by engaging intensively with the data (Alvesson and Kärreman, 2007). Drawing on extant theory as a sensitizing device (Walsham, 2006), we then increasingly refined these tentative insights by scrutinizing the data through coding, categorizing and comparing.

After initial familiarization with the data, three of the authors undertook the more detailed coding process using Nvivo software. They collaboratively drafted a coding scheme that was inclusive of initial empirical insights regarding the importance of cognitive framing and trust processes. Each coder then separately coded the same champion interview, after which they came together to discuss and refine the codebook. This led to revising codes and adding numerous code themes. For the rest of the interviews, the first author acted as the primary coder while the two other authors served as second coders providing inter-coder reliability, suggesting changes and additions to the codes and coding of interview quotes. This process was highly discursive and iterative, and the codebook was revised and refined on multiple occasions during the process. In addition, the coders had in-depth discussions as to how interview quotes should be coded, which led to further refinement in code definitions, code themes and the coding of interview quotes.

## Results

**FIGURE 2:**
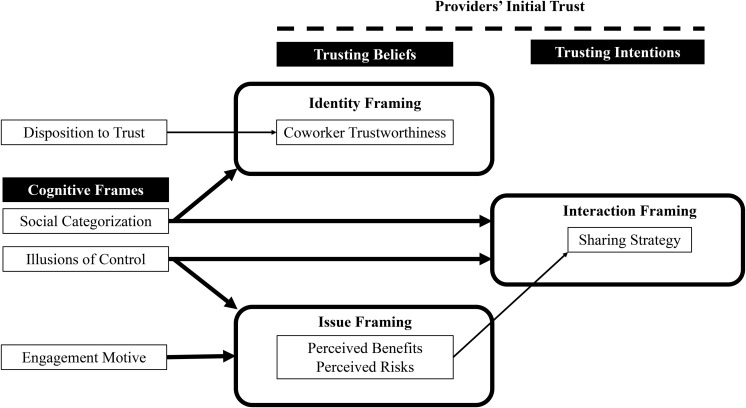
Providers’ initial trust and the framing of collaborative consumption on an organization-sponsored sharing platform.

Given our interest in trust dynamics, we developed and utilized detailed trust codes and themes (see Table 2) to analyze the champion interviews. We then classified each champion as having low, moderate or high initial trust as a provider (see Table 1) using an overall assessment of our trust-related coding of *first* interviews with each of them. This classification enabled us to further theorize the framing of providers’ initial trust. Through our abductive analysis of champion interviews, we developed a conceptual model (see Figure 2) in response to our research questions: *What is the nature of trust among employees who initially provide goods and services on an organization-sponsored sharing platform? How does it influence the way these providers perceive and enact collaborative consumption with coworkers?* Our conceptual model depicts how three types of frames – identity, interaction and issue frames – shape providers’ initial trust, which is comprised of their trusting beliefs and intentions. We now describe the in-depth qualitative insights that we theorized in order to develop our conceptual model. The letter at the end of each interview quote indicates the champion who made those comments (see Table 1).

**TABLE 2:** Trust codes and themes.

Code	Definition
Coworker trustworthiness	Champion’s trusting belief is based on coworker characteristics.
Ability	Related to knowledge, skills, resources and experience.
Benevolence	Related to intentions, motives and interests.
Integrity	Related to values and goals, often based on common organizational membership.
Disposition to trust	Champion’s characteristics that influence their initial trust as providers.
Trusting Stance	Champion consciously decides to trust/not trust coworkers after situational analysis.
Faith in Humanity	Champion is generally trusting/not trusting of others.
Governance	Champion assumes that agreements/policies, insurance and security deposits or the organization will help govern or mediate disputes or deviant coworker behavior.

### Disposition to Trust and Coworker Trustworthiness

Not surprisingly, we found that champions’ disposition to trust influenced their beliefs about coworker trustworthiness. Their coworker trustworthiness encompassed positive expectations of other employees’ ability, benevolence and integrity, which constituted a general perception rather than a specific assumption about an identified individual. Most champions chose to trust unknown coworkers, including those who acknowledged having a high propensity to trust others.

I’m going to trust them because I have no reason not to trust them. (I)

Maybe I’m too trusting but I think it’s okay. (M)

Trust was definitely a big part of it where I assumed everyone would be a good steward of the thing that they would lend because that was my intention. (L)

### Social Categorization: Identity and Interaction Framing

Champions’ identity was framed by whether they perceived themselves and coworkers as being in a community or a marketplace. The latter social categorization also framed how champions made sense of their interactions with coworkers (see Table 3). Those who perceived themselves as being in a community described themselves as prosocial. They considered collaborative consumption to be a culturally normative practice that they engaged in with friends or within their work team. On the other hand, champions who perceived themselves as being in a marketplace described themselves as materialistic. They considered collaborative consumption with coworkers to be an irregular practice that was uncommon or only done occasionally to address discretionary consumption rather than everyday consumer needs. Social categorization, therefore, anchored different meanings of coworker collaborative consumption that were salient for champions and in tension on our organization-sponsored sharing platform.

**TABLE 3:** Identity and interaction framing based on social categorization.

Social categorization	Frame type	Frame theme	Exemplar quote
In community	Identity	**Prosocial:** Discusses collaborative consumption as a relational or communal activity, often involving communication or generosity.	*It kind of forces people to be able to communicate in a way that email doesn’t allow. I mean with this app, you have to talk to someone, you have to coordinate, you have to meet and pickup and drop off … You can go to get what you need, but you also have a bonus experience of seeing someone in person because you’re expecting that’s what you have to do in order to make that exchange happen. (M)*
In community	Interaction	**Normative:** Discusses collaborative consumption as a common practice that they engaged in with friends or within their work team.	*I think we do this informally every day, right? I know my colleague in the [university center]. I can call her and say “Hey, I’m short a power cord.” I stole a power cord from her, right? And she handed that to me or sitting in a meeting and my laptop’s dying someone gives me their power cord, right? (N)*
In marketplace	Identity	**Materialistic:** Discusses collaborative consumption as being about consumer need, trial or experience of goods and services, possibly leading to purchase/ownership.	*What I’m hoping for is kind of to serve as a sort of marketing ad in the sense where, you know, I’d be doing it for them for free on my end. I would treat it as if you were an actual client where you would have to be as professional as possible. And I have to communicate with them effectively and handle deadlines in a serious manner as opposed to just thinking about thinking of it in the sort of sense where oh is this charity. It doesn’t really matter that much. Just because I wanted to save for future purposes where the person next year wouldn’t have the same event come up or they may move on past [the university] and now they work for this big company in the area what not they can think like, “Oh actually I know a guy who’s good at photo shoots let me contact him.” And then that’s where I can tell them, “Last time was free, this time it’s for business purposes.” So this is where we’re going to have to make a contract and exchange money for this. (I)*
In marketplace	Interaction	**Irregular:** Discusses collaborative consumption as something that is infrequent or only done occasionally to address planned needs.	*I don’t think that I’ve gone in with like, “Oh, I need this thing let me see if someone has put it on there.” … I think of this more as like an ongoing thing like if I have a project that I know, you know, in a week’s time or something I’ll need like a saw or whatever then I would come from here to look for that. But I think I just sort of browse more than anything. (L)*

### Engagement Motives and Perceived Benefits: Issue Framing

In discerning other cognitive frames, champions’ primary goal when consuming collaboratively with coworkers was consequential. Champions indicated either a citizen or consumer motive for engaging on our organization-sponsored sharing platform.

Just to help out. (A)

Well because, because I’m always asking for stuff around here, does anybody have this? I need to borrow it. (B)

**FIGURE 3:**
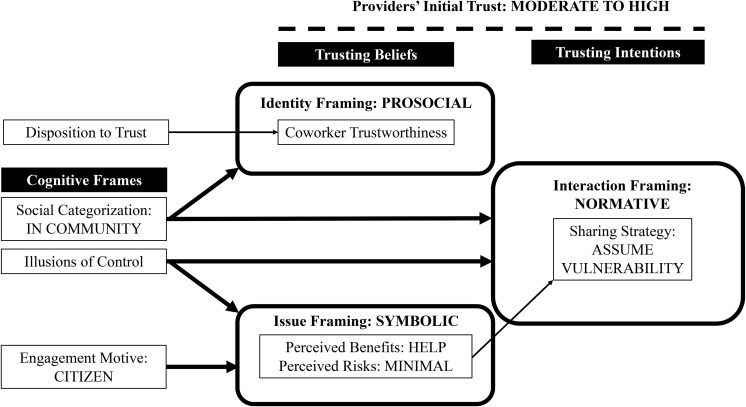
Providers’ initial trust when coworker collaborative consumption is framed as sharing within a community of citizens.

**FIGURE 4:**
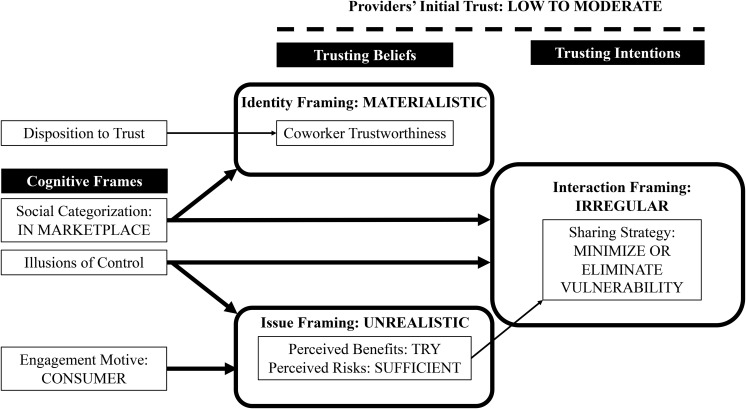
Providers’ initial trust when coworker collaborative consumption is framed as sharing within a marketplace of consumers.

Their engagement motive framed how champions perceived the substantive act of sharing goods and services with coworkers (see Table 4). Those with a citizen motive (see Figure 3) described coworker collaborative consumption as symbolic because it was a meaningful and tangible practice to them or had organizational significance. Champions with primarily a consumer motive (see Figure 4), however, described coworker collaborative consumption as unrealistic because they experienced it as a problematic or infeasible practice.

**TABLE 4:** Issue framing based on engagement motive.

Engagement motive	Frame type	Frame theme	Exemplar quote
Citizen	Issue	**Symbolic:** Discusses collaborative consumption as a meaningful and tangible practice or having organizational significance.	*It makes sense for as an organization to be able to share things and to share resources and time, whatever that looks like. It makes us better as an organization and we know that as an organization we’re better, we’re smarter collectively than we are individually, that kind of thing. (C)*
Consumer	Issue	**Unrealistic:** Discusses collaborative consumption as a problematic or infeasible practice.	*Yeah. It’s not realistic. Yeah. Well if they want it really bad but they’re going to spend 40 bucks in gas to get to my house to pick it up when they could go rent it down at Home Depot probably for the same 40 bucks and not feel obligated or worried about it. (F)*

When evaluating gains, consumption was by far the most important benefit that champions perceived in sharing goods and services with coworkers. However, champions with a consumer motive focused on how coworker collaborative consumption enabled them to try new things whereas those with a citizen motive described how it enabled them to help coworkers. In other words, champions with a citizen motive created value by providing goods and services on the platform in contrast to those with a consumer motive who accrued value by consuming them.

I needed this camera, like a rather good camera, to shoot the presentation for my [course] teams, and I needed a relatively good microphone to record myself giving talks to the students. And those things were not available anywhere … So I just you know spent a few thousand dollars from the grant and bought a couple things. They’re very, very useful and I’m pretty sure that other folks don’t have those. So I figured yeah they might be useful. (E)

I have my own camera here. But the full frame camera is something that I’ve been wanting to get my hands on. I want to shoot a few things and kind of use that as a comparison to what I have now to see if it’s something that I should really invest in. (I)

Regardless of their engagement motive, our organization-sponsored sharing platform enabled champions as providers to help others save or not waste money, as well accrue these benefits themselves as consumers. Interestingly, champions with extra stuff accrued an additional benefit from coworker collaborative consumption because it alleviated underutilization of their excess goods and resources or perceived insufficient appreciation of their belongings, as well as any associated guilt. Some even described how providing goods and services would facilitate their own learning and growth, as well as their coworkers’ acquisition of knowledge and skills. Collaborative consumption also offered friendship benefits for most champions because providing goods and services entailed meeting and connecting with coworkers, which was a source of enjoyment for many who experienced positive emotions when doing so.

I think it’s a great idea for people that have things that just you know sit around a lot that they can be used by somebody else. That’s why I already loaned them out to family and friends, in fact the vases that I have listed are being used Saturday at a wedding because another family through a family member knew I have them and so she borrowed. But I think it’s great because otherwise they just sit around. (K)

So for me is appealing to put that in the service because it’s another opportunity to meet other people. (G)

### Illusions of Control and Perceived Risks: Issue Framing

Champions’ illusions of control also served as a substantive issue frame for evaluating the risks of consuming collaboratively with coworkers. They primarily discussed how lending or borrowing goods meant that their or coworkers’ property was susceptible to wear and tear, damage, and even theft or loss.

Those who perceived minimal risks assumed that coworkers would appreciate and take good care of their belongings. These champions expressed confidence that all employees, including themselves, were acting with goodwill.

I would feel terrible if I had something and something happened to it. You know I returned it to you damaged or you know particularly something that someone may need to use again or someone would have used. So, I think it’s just probably more of a kind of an agreement or whether it’s just a normative piece of what this experience is about. Take care of it as if it was yours. Kind of a social contract more than I think I’ll be going to small claims court. (D)

In contrast, other champions perceived sufficient risks that shared belongings would get damaged during collaborative consumption. These champions’ fears were mitigated by devising token control efforts or assuming that they could track down other employees in the event that their shared item was broken, stolen or lost.

I definitely would say that fear would hold me back a little bit from borrowing until I get more comfortable perhaps using the app. Maybe I would start with like borrowing someone’s Uno game. See what it’s like. (O)

At least I know where they work and can track them down, if it’s an issue with that, or if it comes back messed up or something. So that did offer some comfort. (J)

Irrespective of any illusions of control, champions frequently talked about relationship costs, which centered on how sharing a good or service with coworkers can lead to social awkwardness or tension, loss of privacy, and even negative emotions if property damage occurs, which would also cost them money. Many champions acknowledged that providing goods and services was a commitment of their time and effort. It also limited their own consumption because they could not personally use the goods that they lent out or the time that they spent volunteering services to coworkers.

### Sharing Strategies

Champions who perceived minimal risks in coworker collaborative consumption shared goods and services that were valuable or useful to them. In other words, their trusting intentions reflected a sharing strategy of assumed vulnerability.

If someone said that my working item wasn’t working and then they returned it to me broken. Yeah I would have a concern about that but also people who are typical to lend and borrow typically are respectful of items. And you know I’ve never had someone return something broken without replacing it. (N)

On the other hand, champions who perceived sufficient risks only shared goods that were of little or no value to them, typically extra stuff they had laying around. They did not share anything of value. Their trusting intentions reflected a sharing strategy that minimized or eliminated vulnerability.

Again because the items that I’m selecting are not high value in terms of price and also because they’re not an item that I’m using on a day to day basis, probably wouldn’t be too much of an issue for me if they damaged it. (H)

I have a camera that is like a five thousand dollar camera that I barely get to touch because it’s too expensive and replacing it is gonna hurt. It’s gonna hurt the budget but then also the time that it’s gonna take to lose it. So some of those type of things are things that I that I’m not willing to share. (C)

## Discussion

Our results contribute to the emerging literature on trust in sharing economy (Cho et al., 2007; Belk, 2010) by providing rich, qualitative insights about providers who are an understudied yet critical group of users on sharing platforms (Botsman, 2010; Huurne et al., 2017). Through abductive analysis of 22 interviews with 15 champions, we shed light on how these providers initially develop trust when consuming collaboratively on an organization-sponsored sharing platform. To conceptually ground our data analysis, we first integrated prior research on trust among employees (McKnight et al., 1998; Colquitt et al., 2007) and cognitive framing (Dewulf et al., 2009; Lewicki and Brinsfield, 2011). Our empirical findings reveal how champions in our field study enacted coworker collaborative consumption as citizens in a community or consumers in a marketplace (see Figures 3, 4). Their social categorization and engagement motive together with their illusions of control represented identity, interaction and issue frames that shaped their beliefs about coworker trustworthiness and intended sharing strategy.

When probed on how we could mitigate their perceived risks during *second* interviews with some of them, champions had quite a few suggestions for platform governance. Managers and software developers should consider these recommendations for institution-based trust when designing and implementing digital platforms in the sharing economy. Specifically, champions who perceived themselves as citizens in a community were in favor of adding reputation ratings, agreements and organizational policies.

Yeah, it [ratings] would actually be really cool. I’m very fond of that for Uber, Lyft and the different things. Airbnb is one that I have experience with a lot just because you have to leave ratings for your host and I know they leave ratings for you and they leave comments and things like that. So it would be helpful to be able to see other people vouching for the validity of that person and how they took care of your items. So yeah, that would be a cool feature that could be added. (J)

I think it might be a good idea to have some kind of agreement or something. If you are borrowing something that’s over, I don’t know, five hundred dollars or something, if you have some kind of written statement saying, I understand that I am borrowing this and I understand the value and yes, I intend to take care of it and use it responsibly. I think that that might give a lot of people who may have these high value items to be lent, it might give them a little peace of mind. I’m thinking of how also that might scare people from doing it. So, I don’t know. I feel like that’s kind of a double-edged sword, but yeah that would be the only suggestion that I would have. (M)

What happens if I break it, and then, university policy. So, let’s say I did borrow your department’s projector and I broke it. So, does that mean my department pays for it? (N)

On the other hand, champions who perceived themselves as consumers in a marketplace discussed insurance and security deposits.

So in the sense of using insurance, like if I borrowed someone’s very expensive camera, I would say yes could you please give it to me just in case even though I myself am already comfortably familiar with most cameras. I know the value of them both monetary and the emotional value that someone has with their camera. So I would definitely always opt for getting the insurance. At the same time, if I were to share some of my equipment with someone, I would most definitely want it to be a requirement that they must. If they borrow from me, they must get the insurance. And it’s not necessarily because I wouldn’t trust a person. It’s just more of an extra safety measure, as a just in case. It’s better to have it and not need it. (I)

I had my eyes on that camera. I’m kind of looking at it but I’m also kind of afraid… It said the estimated value is greater than five hundred dollars and so in my mind I’m thinking like am I prepared to replace this item if I break it? And if I were to borrow something like that, I would feel responsible for replacing it. But I’m also kind of weird. So I don’t expect other people to do that for me. But I would feel really bad if I broke someone’s camera. When I was looking at this app that wasn’t my initial thought. I was thinking of that camera. I am really into photography so it’d be cool … I mean as wild as it might sound, I do think having the ability to maybe put a deposit down would help me feel a little bit better. Just saying there’s a bit of security there for myself. I don’t know anything about insurance so I think that also could be a potential option as well. I just have no idea how that would work or what that even looks like. So some type of security that could make me feel a little bit better about the risks that I’m assuming by borrowing someone’s nice item. If I were to message them and they say if it breaks you’re responsible for replacing the entire thing then that probably would make me not want to follow through. Not that I think I’m going to do anything bad to it but I’m just assuming the worst case scenario. (O)

Our findings are consistent with prior research highlighting the challenges of sustaining an organization-sponsored sharing platform (Bhappu and Schultze, 2018). That being said, the inherent novelty of this technology and its facilitated sharing practice in workplaces deserves further investigation given its potential for increasing sustainable consumption. Insights about employees’ cognitive framing and suggestions for platform governance from our study offer potential avenues for overcoming barriers to scaling these two-sided platforms. In particular, future research on sharing platforms should investigate how to engage users who are not only consumers in a marketplace but also citizens in a community. Without enabling consumer market mechanisms, however, sharing goods and services with coworkers on an organization-sponsored sharing platform currently falls into the realm of extra-role employee behavior. If organizations are truly committed to promoting collaborative consumption among employees, they should legitimize it as in-role employee behavior by putting in place the necessary policies, procedures and systems integration (Bhappu and Schultze, 2018) needed to create a local circular economy in workplaces bolstered by community-based, coworker trust. Understanding how to make the sharing of goods and services among coworkers a normative task is key to promoting collaborative consumption and organization-sponsored sharing platforms.

Some employees in our field study had difficulty understanding how the mobile applications worked, as well as identifying goods and services to share with coworkers, although they had access to related information and video tutorials on our study website. An additional limitation was that several Champions 1.0 knew members of the research team who were also university employees. These champions’ interpersonal relationships could have influenced their trust of the platform provider (research team members), or lack thereof. Similarly, low levels of user registrations and activity on our organization-sponsored sharing platform may have reflected an unwillingness on the part of employees to participate in a research study associated with university colleagues.

Future research should explore whether the effects of homophily and perceived similarity on trust and sharing behavior are categorized into “social spheres.” One community champion with a citizen orientation was willing to lend very expensive and useful equipment to other faculty members but was more hesitant about sharing it with staff at the university. Additionally, future research should explore whether and how different types of the collaborative tasks influence trust among employees. It may be that employees are more trusting of coworkers when performing normative work-related tasks than engaging in collaborative consumption, which is currently a novel and discretionary task in most organizations.

## Conclusion

Trust is a crucial antecedent for engagement on sharing platforms because it helps mitigate risks during collaborative consumption. However, the literature on trust in the sharing economy has focused almost exclusively on platforms that broker peer-to-peer rental transactions rather than social exchanges. There is also a lack of research about providers’ perspectives. We addressed these gaps by investigating the nature of trust among employees who initially provide goods and services on an organization-sponsored sharing platform. We also explored how these employees’ initial trust influences their collaborative consumption with coworkers. By integrating prior research on initial trust among employees and cognitive framing with abductive analysis of qualitative interviews, we developed a conceptual model depicting how identity, interaction and issue frames shape providers’ beliefs about coworker trustworthiness and intended sharing strategy. In particular, our empirical findings revealed that employees’ social categorization, illusions of control and engagement motive framed their initial trust and enactment of collaborative consumption as citizens in a community or consumers in a marketplace.

## Data Availability Statement

The datasets generated for this study are available on request to the corresponding author.

## Ethics Statement

Our study was approved by the University’s Institutional Review Board and we obtained consent from participants to share their deidentified (redacted) interview quotes in research reporting and publication.

## Author Contributions

AB designed and conducted the field study, collected the data, and drafted an initial manuscript. KB, TA, PZ, MY, and TL helped to analyze the data and edit the manuscript. All authors contributed to manuscript revision, read and approved the submitted version.

## Conflict of Interest

AB founded and is co-owner of Sharing Tribes LLC, which was the company that developed the sharing platform and mobile applications used in this research study. The University of California, Merced executed a service agreement with Sharing Tribes LLC to license and customize their technology for this research study. The remaining authors declare that the research was conducted in the absence of any commercial or financial relationships that could be construed as a potential conflict of interest.

## Appendix

### Champions 1.0 and 2.0 First Interview

#### Introduction [Only probed with Champions 2.0 who were already platform users.]

Why did you initially register for the apps? What were your expectations?

How would you characterize your engagement so far as an app user?

Why did you agree to serve as a champion?

What do you expect your engagement as an app user to be moving forward, and why?

Please show me the items that you have listed.

#### Probe Offered Goods

Why did you decide to share this good? What considerations led you to lend this good over others?

How meaningful is this good to you? Do you have particular memories associated with it?

What is the value of this good to you? How did you decide the $ value to assign it on the app?

When and where did you first acquire this good? Where does is reside now? How often do you lend out this good?

Who do you think will borrow this good, and why? What will they value about it? How would you feel if no one borrowed it?

What concerns do you have about lending this good? How would you feel if a coworker damaged the good that you lent to them?

#### Probe Offered Services

Why did you decide to share this service? What considerations led you to volunteer this service over others?

How meaningful is this service to you? Do you have particular memories associated with it?

What is the value of this service to you? How did you decide the $ value to assign it on the app?

When and where did you first provide this service? Where do you offer it now? How often do you volunteer this service?

Who do you think will request this service, and why? What will they value about it? How would you feel if no one requested it?

What concerns do you have about volunteering this service? How would you feel if a coworker criticized the service that you provided to them?

#### Probe Posted Needs

What considerations led you to post this need? What is the value of this good/service to you?

How frequently do you need this good/service? How have you addressed this need up to now?

Who do you think will respond to your posted need, and why? How would you feel if no one responds?

What concerns do you have about posting this need? How would you feel if a coworker refused to share the good/service that you posted as a need?

### Champions 2.0 Second Interview

#### Probe Experience

Tell me about your experience using the Share@Home and Share@Work mobile apps to “connect and consume more sustainably” with coworkers.

How frequently did you use the apps? What helped and what hindered your user engagement?

What actions did you take on the apps? What were you hoping to share? Did you succeed?

What issues did you have using the apps? How did they manifest? How did they get resolved?

Was your experience different when you were the provider versus the consumer of shared items?

#### Probe Trust

Did trust play a role in your experience using the apps? How so?

What did you evaluate when deciding whether to trust?

How did trust influence your engagement with the apps over time?

Was trust different when you were the provider versus the consumer of shared items?

How trustworthy were the app users? Why? How about other coworkers?

How trustworthy was the app technology? Why? How about other mobile apps?

#### Probe Sharing

How does sharing goods and services compare to sharing knowledge with coworkers?

Ideally, how would you like to “connect and consume more sustainability” with coworkers?

Should the university continue sponsoring the apps when the research study is done? Why?

Is there any other feedback that you would like to provide?
